# Photopolymerization 3D-Printed Dual-Modal Flexible Sensor for Glucose and pH Monitoring

**DOI:** 10.3390/s25175358

**Published:** 2025-08-29

**Authors:** Shao Lin, Yu Li, Zhenyao Yang, Qiuzheng Li, Bohua Pang, Yin Feng, Jianglin Fu, Guangmeng Ma, Yu Long

**Affiliations:** 1 School of Mechanical Engineering, Guangxi University, Nanning 530004, China; 2201300542@st.gxu.edu.cn (S.L.);; 2 School of Resources, Environment and Materials, Guangxi University, Nanning 530004, China

**Keywords:** biosensors, glucose sensing, pH sensing, flexible materials

## Abstract

Currently, flexible sensors based on electrochemical principles are predominantly limited to single-parameter detection, making it challenging to meet the demand for synchronous monitoring of multiple analytes in complex physiological environments. This study presents a 3D-printed flexible sensor for synchronous glucose/pH detection. Glucose was quantified via H_2_O_2_ oxidation current (GOD-catalyzed reaction), while pH was measured through polyaniline (PANI) resistance changes. The ionogel-based microneedle electrode ensures mechanical robustness. At 0.2 V, optimal signal decoupling was achieved: glucose oxidation current dominates, while PANI’s polarization effect is minimized. Neutral pH minimally affected glucose oxidase (GOD) activity, and low glucose concentrations induced negligible pH interference, ensuring orthogonality. In artificial interstitial fluid, the sensor showed glucose: linear response (0.5–2.5 g·L^−1^, 0.288 μA·mM^−1^·cm^−2^); pH: piecewise-linear sensitivity (0.155 Ω/pH·cm^2^ for pH > 7; 0.135 Ω/pH·cm^2^ for pH < 7). The design enables real-time multiparameter monitoring with high selectivity, addressing current limitations in flexible electrochemical sensors.

## 1. Introduction

As the core device of wearable health monitoring, intelligent medical diagnosis, and human–computer interaction, flexible sensors are showing explosive growth driven by the Internet of Things and big data technology. They are widely used in key fields such as real-time physiological signal acquisition (e.g., ECG, blood glucose, pH), robotic tactile perception, and industrial process monitoring [1,2,3,4,5,6]. Market research shows that the global flexible sensor market continues to expand, and it has shown explosive growth in the past few years. In 2020, despite the pandemic, its shipments increased by 32% to USD 444.7 million [7]. Especially in the field of health monitoring, continuous and accurate perception of key physiological indicators (such as blood glucose and pH) is an important direction for flexible sensors to play their core value in the medical field [8].

Among many physiological indicators, glucose and pH have attracted much attention because of their key indicators of metabolic status, tissue environment, and disease diagnosis [9]. Conventional flexible glucose sensing is used to quantify glucose concentration by generating changes in currents arising from reactions catalyzed by glucose oxidase [10]. Flexible pH sensors, on the other hand, generally use resistance/impedance analysis to measure the conductivity of pH-sensitive materials (e.g., polyaniline, metal oxides) as a function of hydrogen ion concentration [11]. However, traditional single-modal sensors based on epidermal contact (e.g., sweat) often face challenges such as insufficient detection accuracy (e.g., sweat components are susceptible to contamination, evaporation) and poor response stability [12]. In order to improve the accuracy and reliability of glucose detection, microneedle percutaneous glucose sensors came into being [13]. By penetrating the stratum corneum and directly contacting the interstitial fluid, it significantly improves the detection accuracy and response speed; however, such microneedle sensors usually focus on the detection of a single parameter of glucose and cannot achieve in situ and synchronous monitoring with pH or other key physiological indicators [14]. In addition, the need to deploy glucose and pH sensors independently (whether traditional epidermal or microneedling) not only increases the burden and discomfort of the wearer but also leads to signal cross-interference and spatial positioning bias, making it difficult to obtain truly synchronized and position-consistent physiological information [15]. This directly leads to the need for integrated dual-modal (or multimodal) flexible sensors. Recent research has focused on the development of integrated sensors that can detect glucose and pH at the same time, and the feasibility and value of synchronous monitoring have been demonstrated by carefully designing the electrode layout, selecting compatible sensing materials, and introducing an isolation layer [16]. For example, the calf saliva detection sensor developed by Md Ridwan Adib et al. used the original gold electrode for pH measurement and then used the effective in situ pH control method achieved by the interdigitated microelectrode (IDE) to optimize the effect of pH on glucose detection [17]. The rhodium oxide nanocoral sensor designed by Dong et al. can simultaneously monitor glucose concentration and pH value in an alkaline environment through its electrocatalytic activity and pH sensitivity [18]. Seung Yun Oh et al. designed a stretchable sweat sensor that responds to glucose and pH by loading different nanocomposites with gold nanosheet electrodes [15] such sensors generally suffer from material bottlenecks (sensitivity and biocompatibility), process limitations (insufficient accuracy in the integration of complex curved micro-nano structures), high cost (use of precious metals and special nanomaterials), and manufacturing difficulties (strict requirements for multi-layer coating and in situ control processes) [19,20,21]. Therefore, there is an urgent need to develop new sensing platforms with excellent flexibility, high-precision manufacturing, good biocompatibility, and reliable dual functions.

In order to overcome the above bottlenecks, this study proposed a strategy based on ionic gel materials and light-curing 3D printing technology, using the dual-parameter response of current resistance to detect glucose and pH. With its high ionic conductivity, wide electrochemical window, and tunable mechanical properties, ionic gels provide an ideal substrate for dual-modal sensing [22]. Digital Light Processing (DLP) technology, on the other hand, overcomes the limitations of traditional manufacturing in the integration of fine structures such as microneedle arrays through micron-level accuracy (10–50 μm) and the ability to form complex structures [23]. In this study, a high-performance gel substrate was developed by optimizing the ionic liquid/photopolymer (PUA-ACMO) formulation, and the core structure of the microneedle electrode was printed directly on the gel, which significantly simplified the process. However, existing studies have not yet systematically addressed the challenges of DLP-based ionic gel sensors in the simultaneous integration of multiple biological functions (e.g., glucose/pH). By layering the glucose oxidase layer (current response) and the polyaniline pH-sensitive layer (resistance response) on the surface of the microneedle, we achieved a dual-modal mutual interference detection within a certain pH influence range (pH6.86–pH7.6) and prepared a two-modal mutual interference detection with good linear sensitivity (sensitivity to glucose concentration of 0.288 μA·mM^−1^·cm^−2^, pH sensitivity in alkaline environment 0.155 Ω/pH·cm^2^). This strategy opens up a new way to efficiently manufacture low-cost, high-precision flexible multimodal sensors.

## 2. Experimental Methods

### 2.1. Materials and Reagents

Polyethylene glycol (PEG, average molecular weight 1000), isophorone diisocyanate (IPDI, average molecular weight 222.28), polycaprolactone (PCL, average molecular weight 2000), tetrahydrofuran, tin dibutyl dilaurate (DBTDL), hydroxyethyl acrylate (HEA, average molecular weight 116.2), hydroquinone (HQ), acryloylmorpholine (ACMO), 1-ethyl-3-methylimidazolebis(trifluoromethylsulfonyl)imide ([EMIM][TFSI], 97%), trimethylbenzoylphosphine oxide (TPO), glucose oxidase (GOD), polyaniline (PANI), and hydrochloric acid buffer (PBS, pH7.0–pH7.4) were all from Maclean’s Biochemical Technology Co., Ltd. (Shanghai, China); silver chloride paste (Ag/AgCl, Elec-H230) was purchased from Shenzhen Yilaike New Materials Co., Ltd. (Shenzhen, China); and glucose solution was purchased from Huasheng Chemical Reagent Co., Ltd. (Zhengzhou, China). pH buffer was purchased from Shengsheng Test Standard Technology Co., Ltd. (Xiamen, China); Nafion™ membrane solution (D520, 5 wt.%) was purchased from DuPont (DuPont, Wilmington, DE, USA); pentylene glycol fixative solution (SF023, 2 wt.%) was purchased from Isejiu Biotechnology Co., Ltd. (Lianyungang, China); artificial interstitial fluid (SBF) was purchased from Feijing Biotechnology Co., Ltd. (Fuzhou, China); and pH test paper was purchased from Luheng Environmental Technology Co., Ltd. (Hangzhou, China). All chemical reagents were of analytical purity and did not require further purification.

### 2.2. Preparation of Ionic Gel Substrate Materials

#### 2.2.1. Polyurethane Acrylate (PUA) Synthesis

Place polyethylene glycol (PEG, 11 g) was placed in a magnetic stirring heater (HJ-2A, Shandong Brocade Scientific Instrument Co., Ltd., Jinan, China) at 120 °C and stirred for 1 h. Isophorone diisocyanate (IPDI, 4.89 g), polycaprolactone glycol (PCL, 2 g), and tin dibutyl dilaurate (DBTDL, 0.04 g) were subsequently added, and 25 mL of tetrahydrofuran was added as solvent. The mixture was stirred in a magnetic stirring heater at 60 °C for 1.5 h, and then hydroxyethyl acrylate (HEA, 1.162 g) and hydroquinone (HQ, 0.04 g) were added to the system, and the stirring reaction was continued for 40 min to obtain polyurethane acrylate with a molar mass ratio of 11:0.5:5.5 for IPDI, PCL, and PEG (PUA); then, we adjusted the dosage of PEG and PCL to obtain PUA with different ratios (11:1:8, 11:0.5:8, 11:0.5:7, 11:0.5:6, 11:0.5:5.5, 11:0.5:5) and labelled them with PEG-xmmol, which represents the molar mass proportion of PEG.

#### 2.2.2. Ionic Gel Synthesis

The above synthesized PUA was stirred with acryloylmorpholine (ACMO) at 70 °C for 4 h, followed by ventilation evaporation to remove the tetrahydrofuran solvent. The photoinitiator trimethylbenzoylphosphine oxide (TPO, 2 wt.%) was added at room temperature, and after stirring for 30 min, 1-ethyl-3-methylimidazole bis(trifluoromethylsulfonyl)imide ([EMIM][TFSI]) with different mass fractions (40 wt.%, 50 wt.%, 60 wt.%, 70 wt.%, 80 wt.%) was added, and the homogeneous ionic gel resin was obtained by stirring for 30 min, with the ionic liquid ([EMIM][TMIM] was represented by ILx%) used to represent the ionic liquid ([EMIM][TFSI]). Part of the resin was injected into a polytetrafluoroethylene (PTFE) mold and was cured by 405 nm ultraviolet light (3 mW/cm^2^) for 1 min to obtain a colorless transparent ionic gel.

### 2.3. Sensor Model Printing

The ionic gel resin was injected into the liquid tank of the DLP 3D printer (S140, BMF Precision Technology Co., Ltd., Chongqing, China) to ensure that the printing platform was completely submerged in the resin and had no air bubbles remaining. To ensure proper adhesion to the print platform and initial stability, we increased the light intensity for the first layer. The first layer of light intensity was 4 mW/cm^2^, the rest of the light intensity was 3 mW/cm^2^, and the light time was 5 s.

Microneedle array (layer thickness 20 μm) and groove (layer thickness 50 μm) models were converted to SLA format and sectioned. Based on the optimized parameters, the curing time of each layer was controlled within a few seconds. After printing, the uncured resin was washed with absolute ethanol and cured for 5 min under a 3 mW/cm^2^ UV lamp.

### 2.4. Prepare Glucose Sensors and pH Sensors

**Figure 1:**
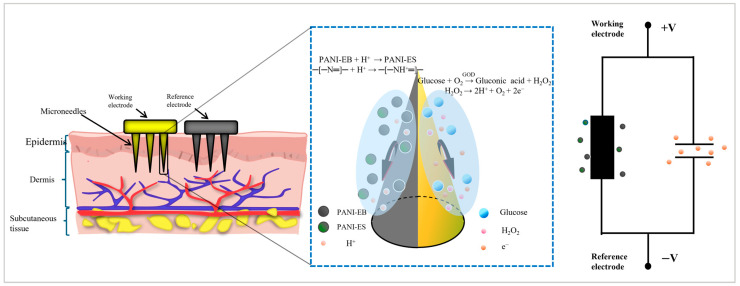
Schematic diagram of the dual-mode sensor working principle.

The sensor operated on two distinct mechanisms: glucose quantification via the oxidation current of hydrogen peroxide (H_2_O_2_), generated through glucose oxidase (GOD)-catalyzed glucose oxidation, and pH detection via the resistance measurement of polyaniline (PANI), whose redox state exhibits pH-dependent conductivity. An ionogel served as the substrate material, with high-resolution light-curing printing employed to fabricate electrode bases featuring microneedle arrays and microgroove structures. The substrate surface was functionalized with GOD and PANI as the glucose- and pH-sensitive elements, respectively. The dual-parameter detection system demonstrated excellent orthogonality, as pH variations in neutral environments minimally affect GOD activity, while the acidic byproducts of glucose oxidation at low concentrations exhibit negligible interference with the PANI-based resistance signal. The working mechanism is illustrated in Figure 1, where pH variation induces a transition in PANI’s state. Under acidic conditions, polyaniline shifts from the emeraldine base form (PANI-EB) to the emeraldine salt form (PANI-ES). The former consists of alternating benzene rings (-C_4_H_6_-) and quinonoid imine rings (=N-C_4_H_6_=N-) with non-protonated nitrogen atoms, while the latter features protonated imine nitrogen, leading to a change in electrical resistance. Meanwhile, glucose is oxidized by GOD, generating H_2_O_2_, and the oxidation reaction releases electrons. The equivalent circuit diagrams for both processes are shown on the right side of Figure 1. To prevent excessive oxidation of PANI by H_2_O_2_, which could impair its pH responsiveness, we explored strategies such as catalase addition, Nafion membrane coating, and pH range adjustment. Ultimately, we utilized the selective permeability of the Nafion membrane to reduce direct contact between H_2_O_2_ and PANI while allowing hydrogen ions (H^+^) to pass through unimpeded. Additionally, we confined the measured pH range to 4–8, which not only aligns with the physiological pH of human interstitial fluid but also mitigates the oxidative reaction between H_2_O_2_ and PANI.

#### 2.4.1. Preparation of Working Electrodes for Glucose Sensors

In this study, two protocols were used to prepare the working electrodes of the glucose sensor. In the first scheme, glucose oxidase (1 wt.%, 5 wt.%, 10 wt.%, 20 wt.%) of different mass fractions was incorporated into the pre-prepared ionic gel resin, and the mixture was stirred for 30 min at room temperature. The microneedle array model was repeated by pouring the enzyme-containing ionic gel resin (5 wt.%) into the liquid tank to obtain a microneedle array with enzymatic activity. After that, the activated copper foil was cut to a suitable size, carefully attached to the bottom or around the groove with tweezers (the ionic gel has a certain stickiness, or it is adhered with an adhesive), and then a certain ionic gel resin was added to the groove, and finally an enzyme-containing microneedle array was placed and cured under 3 mW/cm^2^ ultraviolet lamp for 10 min.

In the second scheme, 0.5 g of glucose oxidase was dissolved in 5 mL of distilled water, mixed with 5 mL of glutaraldehyde solution, and 0.05 mL of mixed droplets were added to the surface of the microneedle array to deposit enzyme membranes. A total of 0.05 mL of Nafion solution diluted with absolute ethanol (1 wt.%) was dropwise applied to the surface of the microneedle array modified with glucose oxidase to form a film precursor, and the mixture was dried at room temperature to form a uniform selective film, with the other encapsulation methods being the same as method 1. The working electrodes prepared by both methods needed to be stored at low temperatures.

#### 2.4.2. Preparation of Working Electrodes for pH Sensors

Polyaniline (PANI, 0.5 g) was dissolved in ethanol–water mixture (mass ratio 7:3), Nafion™ solution (0.5 g, 5 wt.%) was added, the mixture was stirred for 20 min, 0.05 mL was dropwise applied to the surface of the microneedle array, and the mixture was dried at 60 °C for 40 min. The encapsulation method was the same as that of the glucose sensor working electrode.

#### 2.4.3. Preparation of Working Electrodes for Composite pH and Glucose Detection Sensors

Again, two options were used here. The first solution was the same as the method for preparing the pH sensor, first coating a layer of polyaniline and drying for a period of time, and then coating the mixed solution of the second method for preparing the glucose sensor in the first part, and continuing to dry at room temperature for a period of time, which is equivalent to coating two reaction layers.

In the second solution, the first method of preparing the glucose sensor was to prepare a microneedle array containing glucose oxidase, on which a polyaniline reaction layer was coated and dried at room temperature for a period of time. The encapsulation method was the same as the previous method, and the electrodes prepared in both protocols needed to be stored refrigerated.

#### 2.4.4. Preparation of Sensor Reference Electrodes

Silver chloride silver paste (Ag/AgCl) was evenly applied at the bottom of the groove and dried at 100 °C for 40 min, and then a small amount of ionic gel resin was dropped into the microneedle array, and finally, the mixture was cured under a 3 mW/cm^2^ ultraviolet lamp for 10 min to complete the encapsulation.

### 2.5. Material Characterization

FTIR-ATR measurements were performed using a Fourier transform infrared spectrometer (ATR-FTIR (VERTEX 70), Bruker, Karlsruhe, Germany) in the range of 650 to 4000 cm^−1^. We placed 40 wt.%, 50 wt.%, 60 wt.%, 70 wt.%, 80 wt.% ionic liquid ([EMIM][TFSI]) and 40 wt.% ionic liquid ionic gels mixed with glucose oxidase (1 wt.%, 5 wt.%, 10 wt.%, 20 wt.%) of different mass ratios in the above bands to measure transmittance. The surfaces of the six microneedles and the grooves where silver chloride was deposited were observed by light microscopy (CONTOURGT-K, Bruker, Germany) at 100× magnification.

### 2.6. Performance Testing

#### 2.6.1. Mechanical Property Test

The mechanical properties were measured using a universal testing machine (UMT2502, Shenzhen Sansi Zongheng Co., Ltd., Shenzhen, China), and 6 barbell-shaped patterns were prepared for each group of ionic gel samples; the size of the slender part in the middle was 20 mm × 2 mm × 2 mm, and each group was subjected to tensile tests to obtain strain-stress curves, with the better groups selected for cyclic tensile tests. The tensile speed was set to 30 mm/min in the tensile test, the number of cycles was set to 10 times in the cyclic tensile test, and the tensile speed was 10%/s of strain.

#### 2.6.2. Ionic Conductivity Test

Ionic conductivity was measured using electrochemical impedance spectroscopy (EIS) on a digital bridge LCR (TH2832 200 KHz accuracy 0.05%, Changzhou Tonghui Electronics Co., Ltd., Changzhou, China). A barbell-shaped ionic gel sample was sandwiched between two stainless steel electrodes for resistance measurement. The ionic conductivity was calculated according to the following formula:

(1) $$\sigma =\frac{L}{\mathrm{RA}}$$

where L is the electrode spacing, R is the ionic gel resistance, and A is the cross-sectional area of the measurement section.

#### 2.6.3. Finite Element Analysis of Stress–Strain Distribution

Based on the data obtained from the above measurements, the finite element analysis was carried out using COMSOL6.3, and the finite element force analysis was carried out on the individual microneedles and the microneedle array as a whole by establishing a microneedle model. The material parameters were the measured Young’s modulus of 0.12 MPa, Poisson’s ratio of 0.35, etc.; the bottom was set as a fixed constraint, the axial pressure of 1 MPa was applied at the needle end, and the microneedle stress–strain contour diagram under different bottom radius states was plotted. Finally, the bottom radius of the microneedle was determined by analysis to plot the stress–strain diagram of the entire microneedle array under stress.

#### 2.6.4. Sensing Performance Experiments

In the preliminary sensor preparation experiment, the five sensors developed can be divided into three categories according to their functional characteristics, one of which was glucose-specific sensors, including a coated glucose oxidase group and a glucose oxidase embedded group. The second was a pH-specific sensor, including a polyaniline coating group. The third was a glucose/pH dual-function sensor, including a polyaniline and glucose oxidase co-coating group and a polyaniline-coated enzyme-containing microneedle group. The classification system was based on the functional specificity of each sensor and the composition of sensitive materials, in which the dual-function sensor achieved multiple detection capabilities through the synergistic effect of polyaniline (pH response) and glucose oxidase (glucose-specific catalysis). For this purpose, we prepared different concentrations of glucose solutions and different pH solutions, including 1 g·L^−1^, 2.5 g·L^−1^, 5 g·L^−1^, and 10 g·L^−1^ glucose solutions and pH buffers of pH 4, pH 5, pH 6, pH 6.86, and pH 9.18. Samples were taken from each group of sensors, copper foil was pasted on both sides of the bottom, and the two ends were connected to the potentiostat (CH1600E, Shanghai Chenhua Instrument Co., Ltd., Shanghai, China) to measure the current response with the increase in voltage (0 V to 1 V) in different environments.

Glucose-specific sensor sensing performance measurement: 0.02 mL of glucose solutions of different concentrations (increasing concentration) were dropped sequentially, and the current change from the reaction process to the end of the reaction was measured. pH-specific sensor sensing performance measurement: 0.02 mL of buffers of different pHs (from acid to base) were applied sequentially, and the current change from the end of the reaction process to the end of the reaction was also measured. Measurement of the sensing performance of glucose/pH dual-function sensors: The current changes when the above two environmental parameters changed were measured separately. To ensure the dual-modal sensor’s performance under physiologically relevant conditions, we carefully designed the experimental parameters based on the characteristic glucose concentration and pH ranges of ISF. In addition to the original buffer solutions, we incorporated pH 7.2, 7.4, and 7.6 buffers to better represent the typical ISF pH environment (7.35–7.45), while excluding the pH 9.18 buffer to prevent excessive oxidation of PANI by hydrogen peroxide under alkaline conditions. For glucose testing, we prepared solutions with concentrations of 0.5 g·L^−1^, 1.2 g·L^−1^, 1.5 g·L^−1^, and 2.0 g·L^−1^ by diluting 1 g·L^−1^ and 2.5 g·L^−1^ stock glucose solutions with distilled water, covering the physiologically relevant ISF glucose range to ensure comprehensive evaluation of sensor performance across clinically significant concentrations. Then, in order to explore the changes of current and resistance of the composite sensor in the composite environment, on the basis of the original connection to the potentiostat, it was connected to the digital bridge sensor (TH2832 200 KHz accuracy 0.05%, Changzhou Tonghui Electronics Co., Ltd., Changzhou, China), and the bridge was adjusted to DCR mode to measure the resistance, and for the composite materials, it was measured to drop with different pH buffers in turn under the environment of 1 g·L^−1^ glucose concentration (from acid to base) and then 0.02 mL of different glucose concentrations (increasing concentration) were dropped in turn at pH 7.4, and the current peak and resistance were measured. In addition, glucose-pH buffers (1:2, 1:1, 2:1) with different mass ratios were used to measure these parameters at a concentration of 1 g·L^−1^ at pH 7.4. In the application test, a voltage of 0.2 V was applied to a composite sensor coated with GOD and PANI, a certain concentration of glucose solution was added to the SBF every 20 min, the current and resistance were measured, the glucose concentration and pH value were calculated according to the sensing performance, and the corresponding values were measured using a standard blood glucose meter (Yuwell580, Yuyue Medical Equipment Co., Ltd., Suzhou, China) and pH test paper.

The sensitivity of the sensor to glucose concentration was calculated according to the following formula:

(2) $$S=\frac{\text{∆}I_{p}}{\text{∆}C_{\mathrm{gs}}}\;\text{or}\;S=\frac{\text{∆}R_{p}}{\text{∆}C_{\mathrm{gs}}}$$

The sum is the change in the peak value of the current ($\text{∆}I_{p}$) and the peak change in resistance ($\text{∆}R_{p}$), as well as the change in the concentration of glucose ($\text{∆}C_{\mathrm{gs}}$). Sensitivity to pH was calculated according to the following formula:

(3) $$S=\frac{\text{∆}I_{p}}{\text{∆}C_{\mathrm{pH}}}\;or\;S=\frac{\text{∆}R_{p}}{\text{∆}C_{\mathrm{pH}}}$$

Among them, ∆C_pH_. is the change in pH value.

The detection limit (LOD) of the sensor was calculated according to the following formula:

(4) $${LOD}_{gs}=\frac{3\delta }{S_{gs}}\;and\;{LOD}_{pH}=\frac{3\delta }{S_{pH}}$$

The sum is the standard deviation of blank samples (δ) and the sensitivity to changes in glucose concentration ($S_{gs}$), as well as the sensitivity to pH changes ($S_{pH}$). The quantification limit (LOQ) of the sensor was calculated according to the following formula:

(5) $${LOQ}_{gs}=\frac{10\delta }{S_{gs}}\;and\;{LOQ}_{pH}=\frac{10\delta }{S_{pH}}$$

## 3. Results and Discussion

### 3.1. Glucose/pH Dual-Modal Composite Sensor

The structure of skin tissue includes the stratum corneum, epidermis, dermis, and subcutaneous tissue, among which nerve endings are mainly distributed in the junction area between the dermis and subcutaneous tissue. In this study, a 1.6 mm height microneedle array was used, and the penetration depth could accurately reach the interstitial fluid (ISF)-rich area, effectively avoiding nerve stimulation, achieving painless or low-pain detection [24,25]. In order to further improve the detection range and sensitivity, an 8 × 8 microneedle array was designed, which was equipped with a square substrate with a height of 0.8 mm to enhance the mechanical support, and a groove structure (slightly larger than the microneedle distribution area) was constructed on the periphery of the array, which was convenient for the accurate deposition of subsequent electrode materials [26,27,28]. 

For glucose sensors, the basic principle is to apply the electrochemical theory of glucose oxidase. The catalytic reaction of glucose oxidase (GOD) is as follows: glucose + O_2_→glucose acid lactone + H_2_O_2_. The generated hydrogen peroxide undergoes an oxidation reaction on the electrode surface: H_2_O_2_→O_2_ + 2H^+^ + 2e^−^. The current generated by electron transfer has a linear relationship with the glucose concentration. The reaction between glucose and oxygen is catalyzed by glucose oxidase to produce hydrogen peroxide, and the glucose concentration can be detected only by reading the current or resistance change of the modified microneedle array and the corresponding value compared with the glucose concentration value. In the case of pH sensors, the basic principle is the high sensitivity of polyaniline (PANI) to the concentration of hydrogen ions in solution, and the redox state of PANI changes with the change of pH. This process leads to a change in the charge distribution of the PANI conjugate structure, which in turn causes regular fluctuations in the resistance value. Glucose/pH sensors, on the other hand, integrate these two materials into the sensor in different ways [29,30].

**Figure 2:**
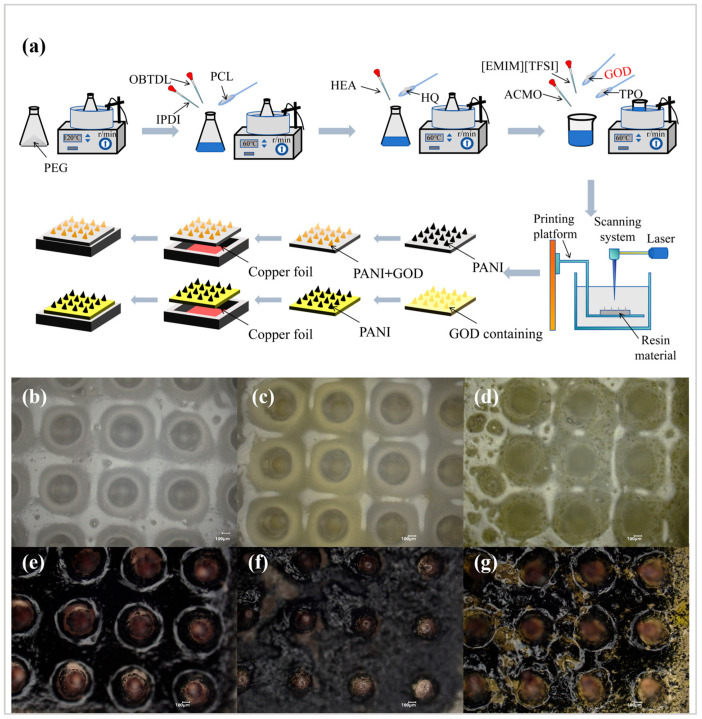
Process preparation and optical microscopy diagram. (**a**) Preparation of glucose/pH composite sensor process. (**b**) Microneedle top view. (**c**) Coated GOD microneedle top view. (**d**) Top view with GOD microneedles. (**e**) Top view of PANI-coated microneedles. (**f**) Top view of coated GOD and PANI microneedles. (**g**) Top view of enzyme-containing microneedles coated with PANI.

The overall experimental flow is shown in Figure 2a. The polyethylene glycol was first dried to prevent the isocyanate (-NCO) from reacting with water to form urea (side reaction), resulting in the consumption of the isocyanate group. The polycondensation of IPDI and polyol, the reaction of isocyanate (-NCO) and the hydroxyl group (-OH) to form a carbamate bond (-NHCOO-), the introduction of HEA and HQ to continue the catalytic reaction while avoiding the self-polymerization of isocyanate to synthesize polyurethane acrylate (PUA), the addition of ACMO and the cross-linking of acrylate groups in PUA to form a three-dimensional network structure, the addition of ionic liquid plasticization, and the addition of glucose oxidase in the process of stirring and mixing can obtain ionic gels containing GOD [31]. Computer-controlled ultraviolet (UV) light selectively irradiates the liquid layer according to the preset layered profile, which is between the ability of TPO to decompose under the action of ultraviolet lamp to produce free radicals and trigger the free radical polymerization of acrylate groups, so that the irradiated area can be quickly cured to form an ionic gel monolayer, and the printing platform descends layer by layer to form a three-dimensional structure. Figure 2a also shows the entire process from coating material to encapsulation of the two GOD/pH composite sensors, while for the working electrode of the glucose-specific sensor, only the glucoenzyme solution needs to be applied or the glucoenzyme-containing microneedles are directly encapsulated as the subsequent steps in Figure 2a. For the working electrode of pH-specific sensors, only the polyaniline solution needed to be coated and encapsulated. For the reference electrode of the sensor, a layer of silver chloride paste was deposited in the groove of an ordinary microneedle and then encapsulated. Figure 2b–g show the top view of the six microneedles under an optical microscope. It can be seen that the deposition of the GOD-coated microneedle array was not high, and only some of the microneedles were successfully modified (the yellow part in Figure 2c is the GOD solution). The degree of microneedle array forming with GOD was not as clear as that of ordinary microneedles. The PANI-coated microneedle array was basically modified (the black part in Figure 2e is a polyaniline solution, which is in powder form after drying). The coating effect of GOD solution was not obvious for the microneedle arrays coated with PANI and GOD. On the other hand, the GOD microneedle array coated with PANI can clearly show the yellow color of the bottom and the part not covered by PANI, and the effect was more obvious. Figures S1 and S2 show the top view of the groove after depositing the Ag/AgCl electrode and the top view under an optical microscope, respectively. Figure S3 shows the working and reference electrodes prepared for each.

### 3.2. Ionic Gel Material Properties

**Figure 3:**
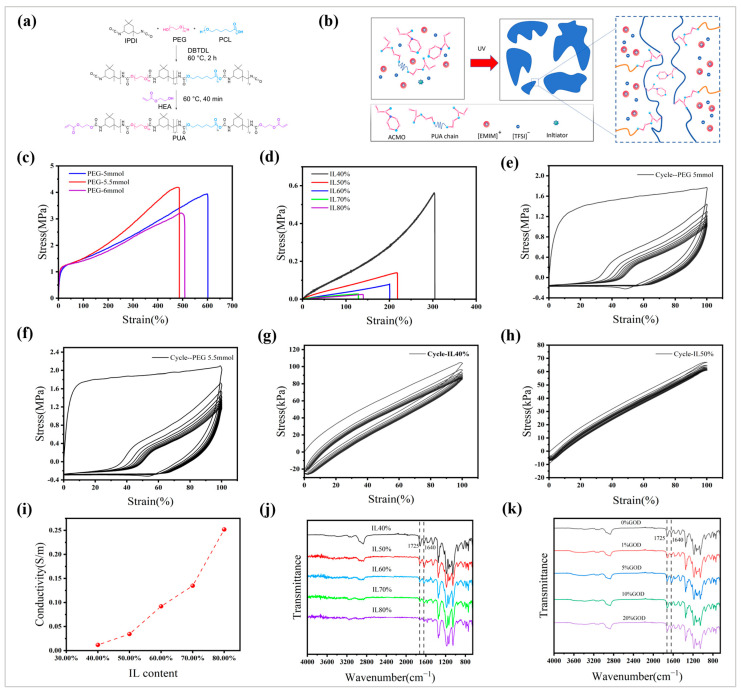
Diagram of ionic gel microstructure and material properties. (**a**) The chemical structure synthesis process of PUA; (**b**) microstructure of ionic gel preparation; (**c**) stress–strain curves of ionogels from PEG-5 mmol to PEG-6 mmol; (**d**) stress–strain curves of ionogels from IL40% to IL80%; (**e**) cyclic tensile curves of PEG-5 mmol under different strains (0–100%); (**f**) cyclic tensile curve of PEG-5.5 mmol under different strains (0–100%); (**g**) IL40% cyclic tensile curve under different strains (0–100%); (**h**) IL40% cyclic tensile curve under different strains (0–100%); (**i**) IL40% to IL80% ionic gel conductivity; (**j**) FTIR spectra of ionic gels with different ionic liquid contents; (**k**) FTIR spectra of ionic gels with different GOD contents.

The photosensitive system consists of acryloylmorpholine (ACMO), polyurethane acrylate (PUA), ionic liquid ([EMIM][TFSI]), and photoinitiator (TPO), where PUA is a UV-curing resin that acts as a chemical crosslinker. Figure 3a illustrates the synthesis process of polyurethane acrylate (PUA). The microstructure of the ionic gel preparation is shown in Figure 3b, where PUA acts as a chemical crosslinker mixed with ACMO, [EMIM][TFSI], and TPO, and the photosensitive system undergoes photoinitiation polymerization under UV illumination [32,33].

The photopolymerization reaction was carried out by the photosensitive system under the irradiation of ultraviolet lamp. By configuring isophorone diisocyanate (IPDI), polycaprolactone glycol (PCL), and polyethylene glycol (PEG) with different molar mass ratios (11:1:8, 11:0.5:8, 11:0.5:7, 11:0.5:6, 11:0.5:5.5, 11:0.5:5), we determined the ionic gel resins that were not prepolymerized and did not have obvious phase separation, and the experimental results showed that the first three groups of gels had obvious phase separation, as shown in Figure S4, and the mass ratio of the last three groups had a better effect, as shown in Figure S5. After the three groups of ionic gels with different PUA to ACMO mass ratios (1:2, 1:1, 2:1) were cured by an ultraviolet lamp, it was found that when the mass ratio was 2:1, the obtained ionic gels had no obvious phase separation and were colorless and transparent, and the hardness of the ionic gels with other mass ratios was too high after curing. In order to further study the mechanical properties of the above materials, we obtained the data as shown in Figure 3c through tensile property tests, in which PEG-5.5mmol had the highest tensile strength of 4.19 MPa, and PEG-5mmol had the highest elongation at break of 617%. The results demonstrated that the PEG-5.5mmol formulation (11:0.5:5.5) exhibited the optimal tensile properties, which was critical for our sensor application due to its requirement for higher tensile strength. PEG-5.5mmol ionic gels were selected to add ionic liquids with different mass ratios. The ionic liquid acted as a plasticizer to reduce the force of the polymer chain and enhance the flexibility of the ionic gel, and it provided a certain electrical conductivity for the ionic gel due to the high mobility of [TFSI] [34]. Figure 3d shows that with the increase in ionic liquid mass fraction, the tensile strength of ionic gels decreased, and the tensile elongation at break showed an overall downward trend, which rose slightly when the ionic liquid mass fraction was 80%, and the maximum tensile strength of IL40% ionic gels was 0.56 MPa, with the maximum elongation at break being 317%. Figure 3e–h show that the cyclic tensile curves of PEG-5mmol, PEG-5.5mmol, IL40%, and IL50% gradually increased from 0% to 100%, respectively. During the loading–unloading cycle, the IL40% and IL50% hysteresis was significantly smaller, demonstrating that the ionic gels exhibited excellent elasticity and stability due to the chemical cross-linking structure. In addition to considering the tensile properties of ionic gels, we investigated the effects of different ionic liquid concentrations on the conductivity of ionic gels. As shown in Figure 3i, with the increasing concentration of ionic liquids, the conductivity of ionic gels continued to increase; this was because the [TFSI]- ions in the ionic liquid [EMIM][TFSI] had a high mobility and formed hydrogen bonds with the polar groups (such as C=O) in the PUA-ACMO network, promoting the formation of ion transport channels. When the mass fraction of the ionic liquid increased from 40% to 80%, the volume proportion of [TFSI]- ions increased, and the density of ion migration paths increased, thereby significantly enhancing the electrical conductivity, and the conductivity of IL40% ionic gels reached 0.012 S/m, which had a certain conductivity. Taking into account both tensile performance and electrical conductivity, we ultimately adopted an IL40% ionic gel resin for sensor fabrication and the light-curing printing of the follow-up experiments.

The FTIR characterization of ionic gels with different ionic liquid concentrations is shown in Figure 3j, where the absorption peaks at 1725 cm^−1^ and 1640 cm^−1^ were attributed to C=O on the PUA and ACMO networks, respectively. As the ionic liquid content increased from 40 wt.% to 80 wt.%, the two absorption peaks narrowed significantly and shifted to a lower wavenumber, indicating that [TFSI]^−^ formed hydrogen bonds with C=O bonds, limiting the vibrational degrees of freedom and promoting the formation of cross-linking networks. As shown in Figure 3k, the infrared characterization of the ionic gel based on 40% IL mixed with GOD with different mass ratios showed that the mixing of different concentrations of GOD did not significantly change the position and width of the absorption peak, indicating that it did not have a chemical reaction with the ionic gel. but only indicated the mixing [35,36].

### 3.3. Microneedle Array DLP Printing and Mechanical Simulation

**Figure 4:**
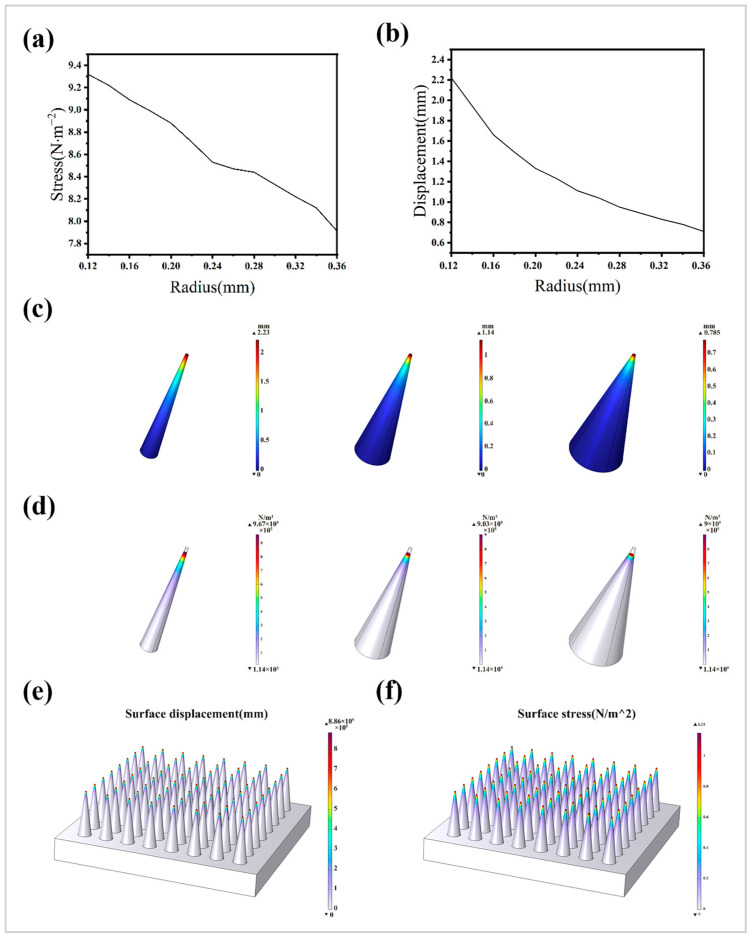
Stress–strain simulation analysis of the microneedle model. (**a**) Stress-radius curve at the top of a single microneedle; (**b**) displacement-radius curve at the top of a single microneedle; (**c**) distribution of microneedle surface displacement clouds with different radii and sizes; (**d**) distribution of microneedle stress clouds with different radii and sizes; (**e**) the overall stress contour of the microneedle array; (**f**) microneedle array overall displacement contour.

Based on the mechanical properties of ionic gel substrate materials, the mechanical simulation of the microneedle array model was carried out in the Solid Mechanics Module [37]. The stress-radius curves and displacement-radius curves of a single microneedle are shown in Figure 4a,b, and the results show that when the bottom radius of the microneedle increased, the tip stress decreased, and the deformation displacement of the top and downward increased gradually. In view of the constraint that the height of the microneedle was fixed at 1.6 mm, it is necessary to balance the contact stress at the top and the deformation amplitude of the structure, so the microneedle with the bottom radius of 0.12 mm, 0.24 mm, and 0.36 mm was selected for comparative analysis. Figure 4c shows the distribution of surface displacement in the same state, when the radius of the bottom of the microneedle increased, the surface stress also tended to decrease and concentrate, but the surface displacement decreased. Figure 4d shows the stress distribution of the three types of microneedles, with the increase in the bottom radius of the microneedles, although the stress peak decreased but the stress was more concentrated at the top. In practical application, we paid more attention to the stress distribution and displacement distribution of the microneedle, although there was still a gap between the Young’s modulus measured by the tensile mechanical properties test and the actual value, but the solid mechanics simulation analysis carried out by the COMSOL6.3 also showed an intuitive result—when the radius of the bottom of the microneedle is too small, the axial pressure generated by the top may cause the surface displacement to exceed its own height and depression. In summary, we chose the bottom radius of 0.24 mm of the microneedles for subsequent DLP printing. Regarding the determination of microneedle spacing, according to Wang et al., the penetration performance of microneedles is negatively correlated with the spacing, and when the spacing exceeds 500 μm, its influence on the penetration depth tends to be flat [38]. Due to the influence of materials and other parameters, the microneedle spacing was determined to be 800 μm, and the model was established based on the above results, and the overall stress and displacement simulations were carried out, as shown in Figure 4e,f.

Before printing microneedle arrays and grooves, the printing parameters of the configured ionic gel needed to be adjusted, and the main parameters that affected the printing effect were the light time of each layer and the intensity of the printing time. The groove needed to have a hollow structure model as a reference, and the model of the hollow small rocket was selected as the model to adjust the printing parameters; because its layer thickness was 50 μm and the number of slices was 50 layers, the printing time was about half an hour, and the efficiency of adjusting the parameters was improved. From the experimental results, it can be seen that when the light intensity and illumination time were large, the model was unable to be hollowed out, and the bottom was still gel adhered and cured, with the forming effect of reducing the light intensity and illumination time being poor. The final printing effect was the hollow rocket model with a light intensity of 4 mW/cm^2^ and the rest of the light intensity of 3 mW/cm^2^ and a illumination time of 5 s. A comparison of hollow rockets with different parameters is shown in Figure S6. Figures S7 and S8 show the microneedle model and groove model printed under this parameter [39].

### 3.4. Sensing Performance Test Results

**Figure 5:**
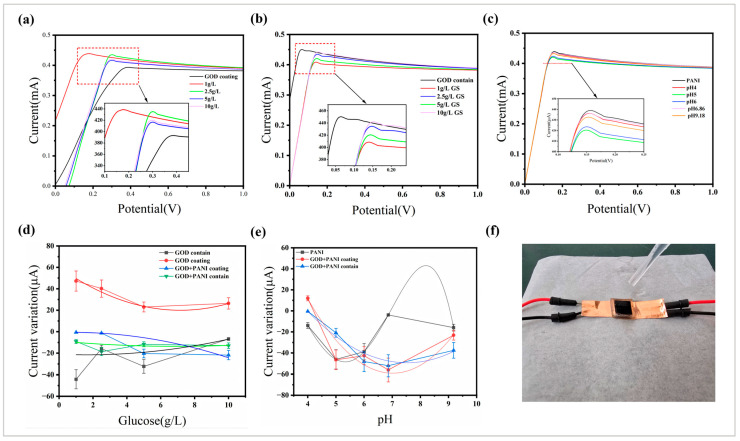
Sensing performance of each sensor in a single environment. (**a**) The magnitude of the current response of the GOD coating sample at different glucose concentrations. (**b**) The magnitude of the current response of the GOD-containing sample at different glucose concentrations. (**c**) The current response of PANI coating samples at different pH environments. (**d**) The change of the peak current of each sample under different glucose concentrations. (**e**) The change in the peak current of each sample at different pH environments. (**f**) The experimental operation demonstration.

Figure 5a–c show the current curve of a sample with a single environmental test capability at different glucose concentrations or pHs. In the electrochemical test of GOD-coated microneedle samples, the peak current increased significantly when 1 g·L^−1^ and 2.5 g·L^−1^ glucose solutions were added, and it decreased when the glucose concentration increased to more than 5 g·L^−1^. This was due to the insufficient amount of enzyme in the microneedles, which made them less sensitive to high concentrations of glucose or the reaction would be complete. The microneedle samples containing GOD also had the most obvious response to the glucose concentration of 1 g·L^−1^, and the peak reduction was the largest, but there was a significant rebound when the glucose concentration increased from 2.5 g·L^−1^ to 5 g·L^−1^, which may have been related to the accumulation of intermediate products in the enzymatic reaction or the inhibition of enzyme activity triggered by the high concentration of substrate. There was no obvious pattern to the current change in the PANI-coated microneedle sample, but the current change occurred for different pH buffers. The above experimental results showed that the microneedle samples coated with GOD had the best current response in a single environment, while the microneedle samples coated with PANI had poor sensing performance.

The relationship between the peak current change and the concentration and pH of glucose solution in a single environment was further studied. To ensure the accuracy of experimental results and data, we performed triplicate measurements for each sample under varying concentrations and pH conditions. The data were further analyzed through curve fitting and error bars to visually represent the preliminary wide-range sensing performance. Figure 5d visually shows the relationship between the change of the peak current and the concentration of the glucose solution. Experimental data show that the GOD-coated microneedles exhibited a significant linear response within the glucose concentration range of 1–5 g·L^−1^ (with a slope of 2.45 μA·mM^−1^·cm^−2^), which is in line with the kinetics of the Michaelis enzymatic reaction—when the glucose concentration was lower than the enzyme saturation concentration, the reaction rate (i.e., the current) was proportional to the substrate concentration. When the concentration exceeded 5 g·L^−1^, the enzyme active center became saturated, and the current growth slowed down or even decreased. The GOD-coated microneedle samples exhibited good linearity at glucose concentrations of 1 g·L^−1^ to 5 g·L^−1^, with a linear response characteristic of 2.45 µA·mM^−1^·cm^−2^ and a linear range of 1 g·L^−1^ to 5 g·L^−1^. The microneedle samples containing GOD had high current sensitivity but a poor linear relationship, which cannot be used as a reference for sensing results. However, the current sensitivity of the composite microneedles to glucose changes in the single glucose environment was low, which may have been due to the current deviation caused by the mutual interference of the two composites, but the current peak change of the microneedle samples coated with GOD and PANI had a monotonically increasing relationship with the glucose concentration, and the linear relationship was better than that of the enzyme-containing microneedles coated with PANI. Figure 5e shows the current peak change versus pH. The GOD microneedle-containing samples coated with PANI had good linearity in the peak change of current in the acidic environment. The current change of the microneedle samples coated with PANI and GOD was basically linear with pH in the acidic environment. There was no obvious pattern between the current change value and pH of the PANI-coated microneedle sample, which may have been due to the inhomogeneity of the PANI-doped state or the irreversibility of protonation/deprotonation [40]. Both types of composite microneedles had high sensitivity in a single pH change environment. The above experimental results show that only the peak current change of the GOD-coated microneedle sample had a linear correspondence with the glucose concentration in the low-concentration environment, while the glucose concentration in the normal human interstitial fluid was generally about 1 g·L^−1^, and the glucose concentration increase after meals is generally about 1.5 g·L^−1^, while the glucose concentration in uncontrolled diabetics will rise to 2 g·L^−1^ to 5 g·L^−1^, so the microneedle sample basically met the function of measuring glucose concentration [41]. However, it is difficult to determine the pH and glucose concentration of the environment by a single parameter change, and the sensitivity was low, with the linearity of the microneedle samples coated with GOD and PANI being relatively better than that of the enzyme-containing microneedles coated with PANI. Based on the fact that the pH variation in human interstitial fluid is relatively limited, the data obtained under single glucose concentration changes more closely approximate the complex physiological environment. Therefore, based on the aforementioned research, we selected the microneedle samples coated with GOD and PANI, which demonstrated better linearity and sensitivity under these conditions, for further investigation. The experimental operation demonstrations are shown in Figure 5f.

**Figure 6:**
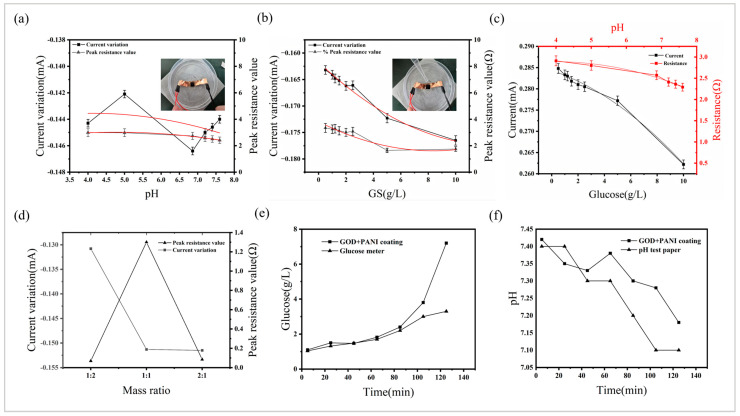
Sensing performance of coated GOD and PANI samples in a composite environment. (**a**) The change of the peak current and the peak resistance of 1 g·L^−1^ glucose concentration with pH. (**b**) The change of the peak current and the peak resistance of the resistance in a pH 7.4 environment with glucose concentration. (**c**) When the voltage was 0.2 V, the current and resistance changed with glucose concentration and pH value, respectively. (**d**) The change in the peak current and the peak resistance under different mass ratios of glucose solutions to pH buffers. (**e**) Comparison of the measured values of the sensor with a standard commercial blood glucose meter in SBF. (**f**) Comparison of the measured values of the sensor and pH test paper in SBF.

We measured the current peak and resistance values at the same time in a composite sample coated with GOD and PANI at a pH-stable environment with glucose concentration and pH at a stable glucose concentration, respectively. The relationship between the peak current and the peak resistance and pH at a glucose concentration of 1 g·L^−1^ is shown in Figure 6a, where the peak resistance decreased with the increase in pH, and the pH value in this environment can be measured according to the corresponding resistance value, with its linear response characteristics shown in the sensitivity of 0.136 Ω/pH·cm^2^ in an acidic environment, 0.422 Ω/pH·cm^2^ in an alkaline environment, and with the linear range being pH 4 to pH 7.6. For pH sensing, the resistance change rate of PANI-coated micrneedles in an alkaline environment was significantly higher than that in an acidic environment, which is related to the protonation equilibrium constant (pKa ≈ 4.5) of PANI at different pH values. Under alkaline conditions, proton dissociation was more thorough, resulting in a more significant change in the charge distribution of the conjugated structure. Figure 6b shows that the peak current change and glucose concentration in the pH 7.4 environment had a good linearity with the glucose concentration, indicating that the acidic environment may optimize the GOD activity and stabilize the PANI conductivity state, and the glucose concentration value in this environment can be measured according to the corresponding current change value, with the linear response characteristic of the sensor being 0.284 μA·mM^−1^·cm^−2^ with a linear range of 0.5 g·L^−1^ to 10 g·L^−1^. However, when the glucose concentration and pH of the environment changed at the same time, after several measurements, we found that the applied voltage of 0.2 V had the best linear response to glucose concentration and pH, and the effects of pH 7 ± 1 on the current and the resistance of glucose concentration in the range of 0.5 g·L^−1^ to 2.5 g·L^−1^ were small (±50 nA). The relationship between the current value and the glucose concentration and the resistance value and pH at 0.2 V is shown in Figure 6c. By analyzing the signal responses in the complex environment, it was found that when the glucose concentration was within the range of 0.5–2.5 g·L^−1^ and the pH was within the range of 6.86–7.6, the cross-interference rate of the two parameters was less than ±20%. This rule stems from the electrochemical window separation of the two sensing mechanisms—the optimal potential of glucose oxidation (0.4–0.6V) and the pH response potential of PANI (−0.2–0.2V) formed the minimum overlapping area at 0.2V, thereby achieving signal decoupling. Furthermore, the high ionic conductivity (0.012 S/m) of the ionic gel substrate provided an independent transmission path for the two-parameter signal, further reducing cross-interference. The response current decreased linearly with the increase in glucose concentration, and the linear response characteristics of the sensor were 0.288 μA·mM^−1^·cm^−2^, with a linear range of 0.5 g·L^−1^ to 10 g·L^−1^. The signal crosstalk effect was less affected by 0.5 g·L^−1^ to 2.5 g·L^−1^. The linear response characteristics of the resistance decreased linearly with the increase in pH, and the linear response characteristics were 0.135 Ω/pH·cm^2^ in an acidic environment and 0.155 Ω/pH·cm^2^ in an alkaline environment, with the linear range being pH 4 to pH 7.6, and the range of signal crosstalk was less at pH 6.86 to pH 7.6. The microneedle was tested in distilled water (0 g/L glucose, pH 7) with 10 replicate measurements of current and resistance, yielding standard deviations of 40 nA and 3 mΩ, respectively, for the blank samples. Based on these measurements, the detection limit (LOD) and quantification limit (LOQ) for glucose sensing were calculated as 0.416 mM·cm^2^ and 1.389 mM·cm^2^, while the LOD and LOQ for pH detection under acidic conditions were determined to be 0.067 pH·cm^2^ and 0.222 pH·cm^2^, and those under alkaline conditions were 0.058 pH·cm^2^ and 0.193 pH·cm^2^. This confirms that our bimodal sensor exhibited minimal parameter interference in neutral pH environments and at low glucose concentrations, providing more accurate measurements for both glucose concentration and pH value, which were closer to the true values. Unlike traditional bimodal sensors designed to avoid pH-induced inaccuracies in glucose measurement, our sensor aims to accurately and simultaneously measure glucose concentration and pH, offering a more comprehensive and precise assessment of metabolic status. Moreover, the measured ranges fell within the typical glucose concentration and pH levels of human interstitial fluid, demonstrating significant potential for applications in biomedical instrumentation requiring accurate dual-parameter monitoring. The bimodal sensor demonstrated a relatively high sensitivity in glucose monitoring. Compared with the high-density silicon microneedle array patch developed by Muamer Dervisevic’s team (with a sensitivity of 0.1622 μA·mM^−1^·cm^−2^), it showed better sensitivity. Although its sensitivity is lower than that of the glucose sensor based on nanomaterials developed by Rafiq Ahmad’s team (which reaches up to 108.15 μA·mM^−1^·cm^−2^), the low-cost flexible materials it uses provide innovative ideas for preparation and application [24,42]. In terms of pH measurement sensitivity, the bimodal sensor was lower than the patch-type sensor studied by Mick Iversen’s team (with a sensitivity of 7.3 Ω/pH·cm^2^) [43]. However, it has the ability to simultaneously measure two parameters, namely, glucose and pH, and its preparation process and materials are simpler. In order to verify the effect of the mass ratio of glucose solution to pH buffer in the composite environment on the experimental results, we took 1 g·L^−1^ glucose solution and pH 4 (the mixture of pH 7.4 buffer solution and 1 g·L^−1^ glucose solution at different mass ratios showed no significant change trend) buffered with different mass ratios to continue to measure the above parameters. The results are shown in Figure 6d. When the mass ratio was close to 1:1, the current peak change and resistance peak value were larger, and the sensitivity was the best, which may be related to the ionic strength balance and the improvement of interfacial wettability. This provides a synergistic optimization direction for multi-parameter sensing designs [44].

In order to further study its application, we placed the sensor in artificial interstitial fluid (SBF) and applied a constant voltage of 0.2 V, added a small amount of glucose solution (5 g·L^−1^) to SBF at intervals (20 min), simulated the scene of glucose concentration rising after meals, measured its current and resistance, and calculated its glucose concentration and pH value from the previously obtained data. At the same time, glucose concentration was measured using a commercially standard blood glucose meter, and pH value was measured with pH test paper. The results are shown in Figure 6e and Figure 6f, respectively, and it can be seen that the accuracy and linearity of glucose concentration measurement were good within 2 h, which is basically the same as that of commercial standard blood glucose meters, but the data was completely distorted due to the enzyme reaction in about 2 h. The pH value was basically stable in the range of 7.1 to 7.4 and changed linearly, and the various ions present in SBF may affect the resistance change and produce certain errors.

## 4. Conclusions

In this study, a flexible microneedle patch with integrated glucose and pH detection functions was developed based on light-curing printing technology, which realized the simultaneous monitoring of multiple parameters in complex physiological environments and solved the problems of high-precision preparation of complex microstructures and poor mechanical properties of materials. However, there are still limitations: while finite element analysis provided theoretical insights into microneedle mechanics, real penetration performance on skin-like materials remains unvalidated. Additionally, morphological changes of microneedles before and after use, as well as the impact of structural damage on sensing performance, have not been experimentally characterized. Other existing issues include low measurement accuracy, limited measurement range, and time constraints. Compared with the traditional preparation methods of microneedle lithography and casting, the one-time molding technology of light-curing printing greatly reduces the preparation cost and complexity. By adjusting the ratio of the photosensitive system, the ionic gels with good mechanical strength (0.56 MPa), breaking strain (317%), and ionic conductivity (0.12 mS/cm) were fabricated, and five samples were printed with good topology and modified for electrochemical measurements. In the single glucose environment, the current peak change of the microneedles coated with glucose showed a good linearity to the glucose concentration, and the sensitivity of the glucose concentration below 5 g·L^−1^ was 0.2 μA·mM^−1^·cm^−2^. When a voltage of 0.2 V was applied to the sensor in the combined environment of glucose and pH, the current of the sample coated with polyaniline and glucose oxidase had a linear relationship with glucose, and the resistance had a linear relationship with the pH in both acidic and alkaline environments, and it decreased monotonically; the mutual anti-interference ability of the two parameters was strong in a certain range under this voltage: the sensitivity of 0.5 g·L^−1^–2.5 g·L^−1^ to glucose concentration was 0.288 μA·mM^−1^·cm^−2^, which had a high sensitivity to alkaline environments (0.155 Ω/pH·cm^2^) and low sensitivity to acidic environments (0.135 Ω/pH·cm^2^), with an effective range of pH 6-pH 8. Based on the development of flexible photosensitive resins and the high-precision and high-integration manufacturing approach provided by light-curing technology, multi-modal flexible sensors will develop in the direction of multi-functional integration, self-energy supply, and biocompatibility in the field of wearable health detection in the future. Future work will focus on conducting penetration tests on skin-mimicking phantoms (e.g., hydrogels with mechanical properties matching human skin) and using SEM to characterize microneedle morphology evolution, thereby establishing correlations between structural integrity and sensor performance.

## Data Availability

The raw data supporting the conclusions of this article will be made available by the authors on request.

## Supplementary Materials

The following supporting information can be downloaded at: https://www.mdpi.com/article/10.3390/s25175358/s1. Figure S1. Top view of the groove after depositing the Ag/AgCl electrode. Figure S2. Top view of the groove where the Ag/AgCl electrode was deposited at 100× magnification under an optical microscope. Figure S3. The working electrode and reference electrode of the sensor prepared for each sample. (a) Reference electrode; (b) working electrodes of enzyme-containing microneedle sensors; (c) coated with the working electrode of the GED enzyme microneedle sensor; (d) coated with polyaniline microneedle sensor working electrode; (e) coated with polyaniline-containing enzyme microneedle sensor working electrode; (f) coated with GED enzyme and polyaniline microneedle sensor working electrodes. Figure S4. Ionic gels with obvious phase separation (IPDI:PCL:PEG = 11:1:8, 11:0.5:8, 11:0.5:7). Figure S5. Ionic gels without phase separation (IPDI:PCL:PEG = 11:0.5:6, 11:0.5:5.5, 11:0.5:5). Figure S6. Comparison of hollow rocket models with different parameters. (a) Finished model with light intensity of 8 mW/cm^−2^ and time of 12 s. (b) Finished model with light intensity of 8 mW/cm^−2^ and time of 10 s. (c) Finished product of the model under the parameters of light intensity of 8 mW/cm^−2^ and time of 6 s. (a) Finished product of the model under the parameters of light intensity of 8 mW/cm^−2^ and time of 4 s. (d) The finished model under the parameters of light intensity 6 mW/cm^−2^ and time 6 s. (e) The finished product of the model with a light intensity of 4 mW/cm^−2^ and a time of 6 s. Figure S7. Actual drawing of the microneedle model. Figure S8. Actual drawing of the groove model.

## Abbreviations

The following abbreviations are used in this manuscript: polyaniline (PANI); glucose oxidase (GOD); digital light processing (DLP); interdigitated microelectrode (IDE); polyethylene glycol (PEG); isophorone diisocyanate (IPDI); polyethylene lactone glycol (PCL); tin dibutyl dilaurate (DBTDL); hydroxyethyl acrylate (HEA); hydroquinone (HQ); acryloylmorpholine (ACMO); 1-ethyl-3-methylimidazolebis(trifluoromethylsulfonyl)imide ([EMIM][TFSI]); trimethylbenzoylphosphine oxide (TPO); hydrogen peroxide (H_2_O_2_).

## References

[1] Ban S., Yi H., Park J., Huang Y., Yu K.J., Yeo W.H. (2025). Advances in Photonic Materials and Integrated Devices for Smart and Digital Healthcare: Bridging the Gap Between Materials and Systems. Adv. Mater..

[2] Yu D., Zheng Z., Liu J., Xiao H., Huangfu G., Guo Y. (2021). Superflexible and lead-free piezoelectric nanogenerator as a highly sensitive self-powered sensor for human motion monitoring. Nano-Micro Lett..

[3] Qu J., Cui G., Li Z., Fang S., Zhang X., Liu A., Han M., Liu H., Wang X., Wang X. (2024). Advanced flexible sensing technologies for soft robots. Adv. Funct. Mater..

[4] Zhou W., Du Y., Chen Y., Zhang C., Ning X., Xie H., Wu T., Hu J., Qu J. (2025). Bioinspired Ultrasensitive Flexible Strain Sensors for Real-Time Wireless Detection of Liquid Leakage. Nano-Micro Lett..

[5] Fan S., Meng L., Dan L., Zheng W., Wang X. (2018). Polymer microelectromechanical system-integrated flexible sensors for wearable technologies. IEEE Sens. J..

[6] Yi Y., Liao H., Liu E., Ye Y., Cai Z., Li X., Li C. (2023). Review of Flexible Biomedical Sensors: Design, Application, and Challenge. IEEE Sens. J..

[7] Yuan Y., Liu B., Li H., Li M., Song Y., Wang R., Wng T., Zhang H. (2022). Flexible wearable sensors in medical monitoring. Biosensors.

[8] Khan S., Ali S., Bermak A. (2019). Recent developments in printing flexible and wearable sensing electronics for healthcare applications. Sensors.

[9] Xiao Y., Xu K., Zhao P., Ji L., Hua C., Jia X., Wu X., Diao L., Zhong W., Lyu G. (2025). Microgels sense wounds’ temperature, pH and glucose. Biomaterials.

[10] Rishpon J., Gottesfeld S., Campbell C., Davey J., Zawodzinski T.A. (1994). Amperometric glucose sensors based on glucose oxidase immobilized in Nafion. Electroanalysis.

[11] Li Y., Mao Y., Xiao C., Xu X., Li X. (2020). Flexible pH sensor based on a conductive PANI membrane for pH monitoring. RSC Adv..

[12] Tang Y., Gan S., Zhong L., Sun Z., Xu L., Liao C., Lin K., Cui X., He D., Ma Y. (2022). Lattice proton intercalation to regulate WO_3_-based solid-contact wearable pH sensor for sweat analysis. Adv. Funct. Mater..

[13] Sharifuzzaman M., Do Shin Y., Yoo J., Reza M.S., Park J.Y. (2023). An oxygen-insensitive and minimally invasive polymeric microneedle sensor for continuous and wide-range transdermal glucose monitoring. Talanta.

[14] Ming T., Lan T., Yu M., Duan X., Cheng S., Wang H., Deng J., Kong D., Yang S., Shen Z. (2024). A novel electrochemical microneedle sensor for highly sensitive real time monitoring of glucose. Microchem. J..

[15] Oh S.Y., Hong S.Y., Jeong Y.R., Yun J., Park H., Jin S.W., Lee G., Oh J.H., Lee H., Lee S.-S. (2018). Skin-attachable, stretchable electrochemical sweat sensor for glucose and pH detection. ACS Appl. Mater. Interfaces.

[16] Li Y., Luo S., Gui Y., Wang X., Tian Z., Yu H. (2023). Difunctional Hydrogel Optical Fiber Fluorescence Sensor for Continuous and Simultaneous Monitoring of Glucose and pH. Biosensors.

[17] Adib M.R., Barrett C., O’Sullivan S., Flynn A., McFadden M., Kennedy E., O’Riordan A. (2025). In situ pH-Controlled electrochemical sensors for glucose and pH detection in calf saliva. Biosens. Bioelectron..

[18] Dong Q., Huang Y., Song D., Wu H., Cao F., Lei Y. (2018). Dual functional rhodium oxide nanocorals enabled sensor for both non-enzymatic glucose and solid-state pH sensing. Biosens. Bioelectron..

[19] He Y., Xu X., Xiao S., Wu J., Zhou P., Chen L., Liu H. (2024). Research progress and application of multimodal flexible sensors for electronic skin. ACS Sens..

[20] Gong X., Huang K., Wu Y.H., Zhang X.S. (2022). Recent progress on screen-printed flexible sensors for human health monitoring. Sens. Actuators A Phys..

[21] Chai J., Wang X., Li X., Wu G., Zhao Y., Nan X., Xue C., Gao L., Zheng G. (2024). A dual-mode pressure and temperature sensor. Micromachines.

[22] Cui X., Xi Y., Tu S., Zhu Y. (2024). An overview of flexible sensors from ionic liquid-based gels. TrAC Trends Anal. Chem..

[23] Petrová E., Chvíla S., Štěpánek F., Zbytovská J., Lamprou D.A. (2025). Imiquimod nanocrystal-loaded dissolving microneedles prepared by DLP printing. Drug Deliv. Transl. Res..

[24] Dervisevic M., Alba M., Yan L., Senel M., Gengenbach T.R., Prieto-Simon B., Voelcker N.H. (2022). Transdermal electrochemical monitoring of glucose via high-density silicon microneedle array patch. Adv. Funct. Mater..

[25] Liu J., Liu J., Liang Y., Yang J., Lin Y., Li Y. (2024). Microneedle-Based Electrochemical Array Patch for Ultra-Antifouling and Ultra-Anti-Interference Monitoring of Subcutaneous Oxygen. Anal. Chem..

[26] Chang K.T., Shen Y.K., Fan F.Y., Lin Y., Kang S.C. (2020). Optimal design and fabrication of a microneedle arrays patch. J. Manuf. Process..

[27] Li R., Zhang L., Jiang X., Li L., Wu S., Yuan X., Cheng H., Jiang X., Gou M. (2022). 3D-printed microneedle arrays for drug delivery. J. Control. Release.

[28] Tariq N., Ashraf M.W., Tayyaba S. (2022). A review on solid microneedles for biomedical applications. J. Pharm. Innov..

[29] Mano N. (2019). Engineering glucose oxidase for bioelectrochemical applications. Bioelectrochemistry.

[30] Beygisangchin M., Abdul Rashid S., Shafie S., Sadrolhosseini A.R., Lim H.N. (2021). Preparations, properties, and applications of polyaniline and polyaniline thin films—A review. Polymers.

[31] Zhang M., Yu R., Tao X., He Y., Li X., Tian F., Huang W. (2023). Mechanically robust and highly conductive ionogels for soft ionotronics. Adv. Funct. Mater..

[32] Alavarse A.C., Frachini E.C.G., da Silva R.L.C.G., Lima V.H., Shavandi A., Petri D.F.S. (2022). Crosslinkers for polysaccharides and proteins: Synthesis conditions, mechanisms, and crosslinking efficiency, a review. Int. J. Biol. Macromol..

[33] Elkhoury K., Zuazola J., Vijayavenkataraman S. (2023). Bioprinting the future using light: A review on photocrosslinking reactions, photoreactive groups, and photoinitiators. SLAS Technol..

[34] Kaur G., Kumar H., Singla M. (2022). Diverse applications of ionic liquids: A comprehensive review. J. Mol. Liq..

[35] Xu F., Wu Z., Tan C., Liao Y., Wang Z., Chen K., Pan A. (2024). Fourier Ptychographic Microscopy 10 Years on: A Review. Cells.

[36] Dubey M.K., Zehra A., Aamir M., Meena M., Ahirwal L., Singh S., Shukla S., Upadhyay R.S., Bueno-Mari R., Bajpai V.K. (2017). Improvement strategies, cost effective production, and potential applications of fungal glucose oxidase (GOD): Current updates. Front. Microbiol..

[37] Shu W., Heimark H., Bertollo N., Tobin D.J., O’Cearbhaill E.D., Annaidh A.N. (2021). Insights into the mechanics of solid conical microneedle array insertion into skin using the finite element method. Acta Biomater..

[38] Wang W., Liang Y., Yan X., Tang G., Xu F., Li Z. (2024). Based on Finite Element Simulation: Optimization of Microneedle Structure and Mechanical Performance Analysis. J. Phys. Conf. Ser..

[39] Mancha Sánchez E., Gómez-Blanco J.C., López Nieto E., Casado J.G., Macías-García A., Díaz Díez M.A., Carrasco-Amador J.P., Martín D.T., Sánchez-Margallo F.M., Pagador J.B. (2020). Hydrogels for bioprinting: A systematic review of hydrogels synthesis, bioprinting parameters, and bioprinted structures behavior. Front. Bioeng. Biotechnol..

[40] Longo G.S., Szleifer I. (2016). Adsorption and protonation of peptides and proteins in pH responsive gels. J. Phys. D: Appl. Phys..

[41] Martins A.J., Velásquez R.J., Gaillac D.B., Santos V.N., Tami D.C., Souza R.N., Osorio F.C., Fogli G.A., Soares B.S., do Rego C.G. (2024). A comprehensive review of non-invasive optical and microwave biosensors for glucose monitoring. Biosens. Bioelectron..

[42] Ahmad R., Lee B.I. (2024). Facile fabrication of palm trunk–like ZnO hierarchical nanostructure–based biosensor for wide-range glucose detection. Chem. Eng. J..

[43] Iversen M., Monisha M., Agarwala S. (2021). Flexible, wearable and fully-printed smart patch for pH and hydration sensing in wounds. Int. J. Bioprinting.

[44] Lan Q., Bassi A.S., Zhu J.X.J., Margaritis A. (2021). A modified Langmuir model for the prediction of the effects of ionic strength on the equilibrium characteristics of protein adsorption onto ion exchange/affinity adsorbents. Chem. Eng. J..

