# Supplementary material for: Photopolymerization 3D-Printed Dual-Modal Flexible Sensor for Glucose and pH Monitoring
Source: Sensors (Basel). 2025 Aug 29;25(17):5358. doi: 10.3390/s25175358 (PMC12431340; doi:10.3390/s25175358)
Supplement: Supplementary file 1 [file sensors-25-05358-s001.zip › sensors-3783688-supplementary.pdf]

Supplementary Materials

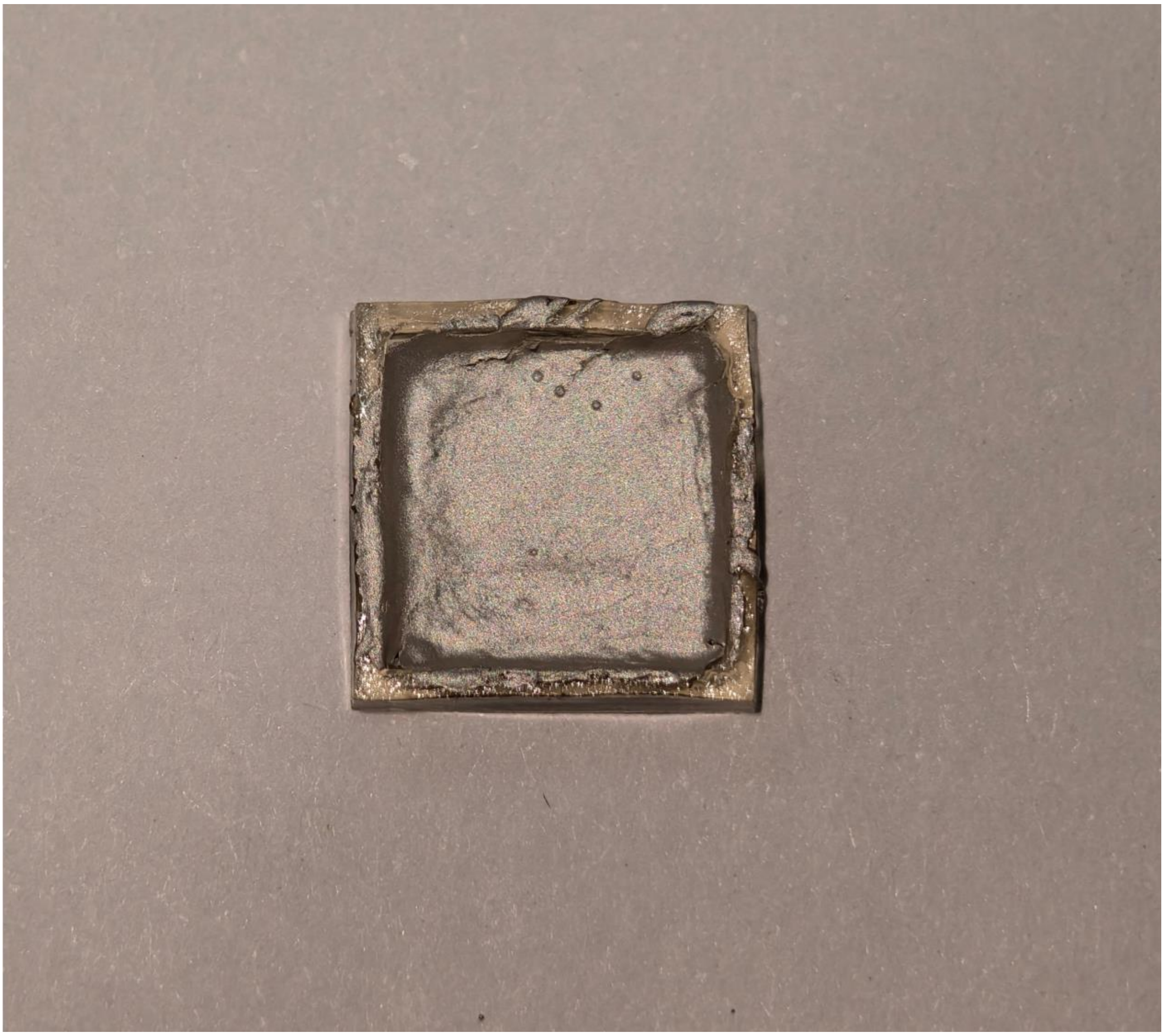

Figure S1. Top view of the groove after depositing the Ag/AgCl electrode

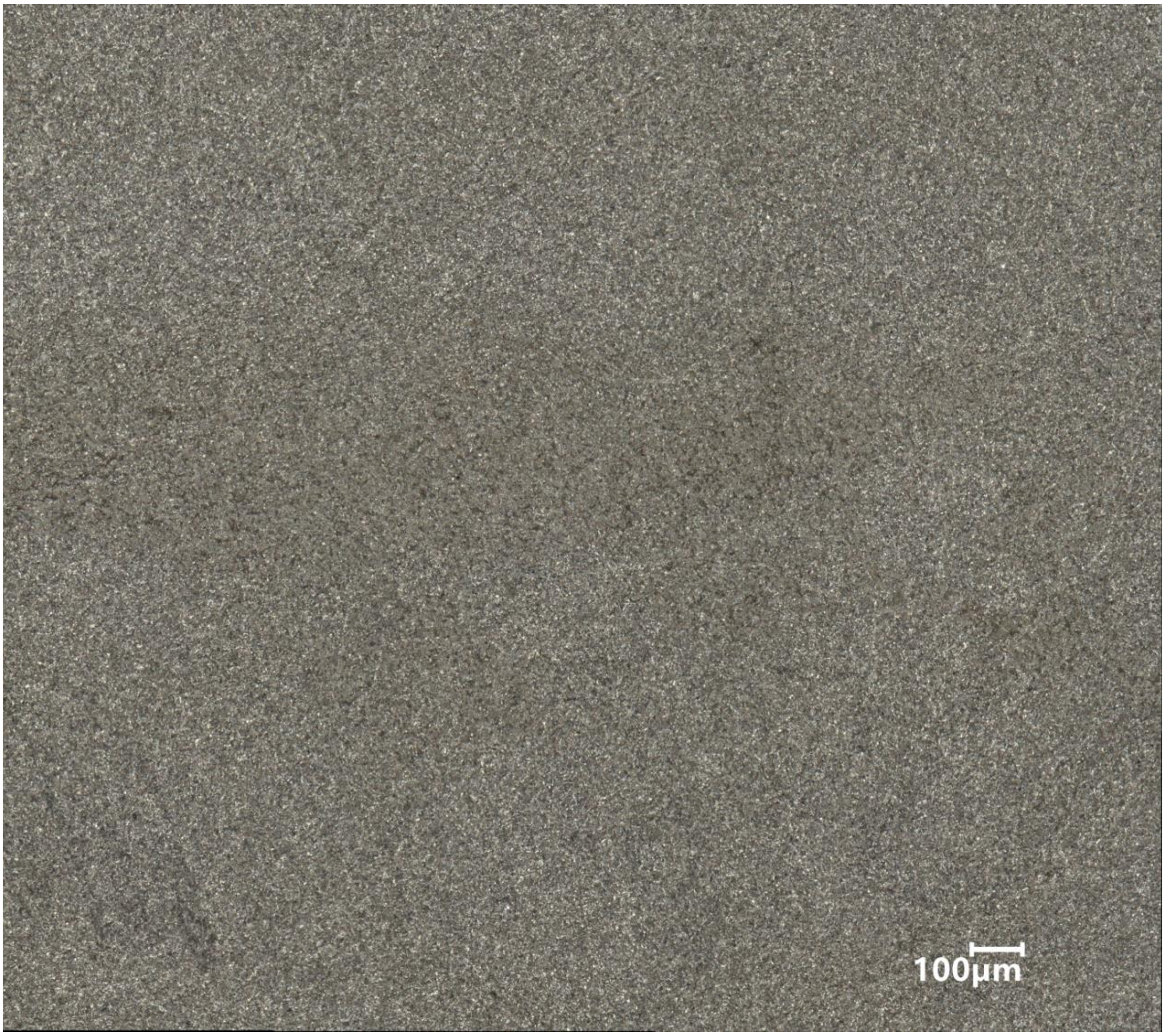

Figure S2. Top view of the groove where the Ag/AgCl electrode is deposited at 100x magnification under an optical microscope

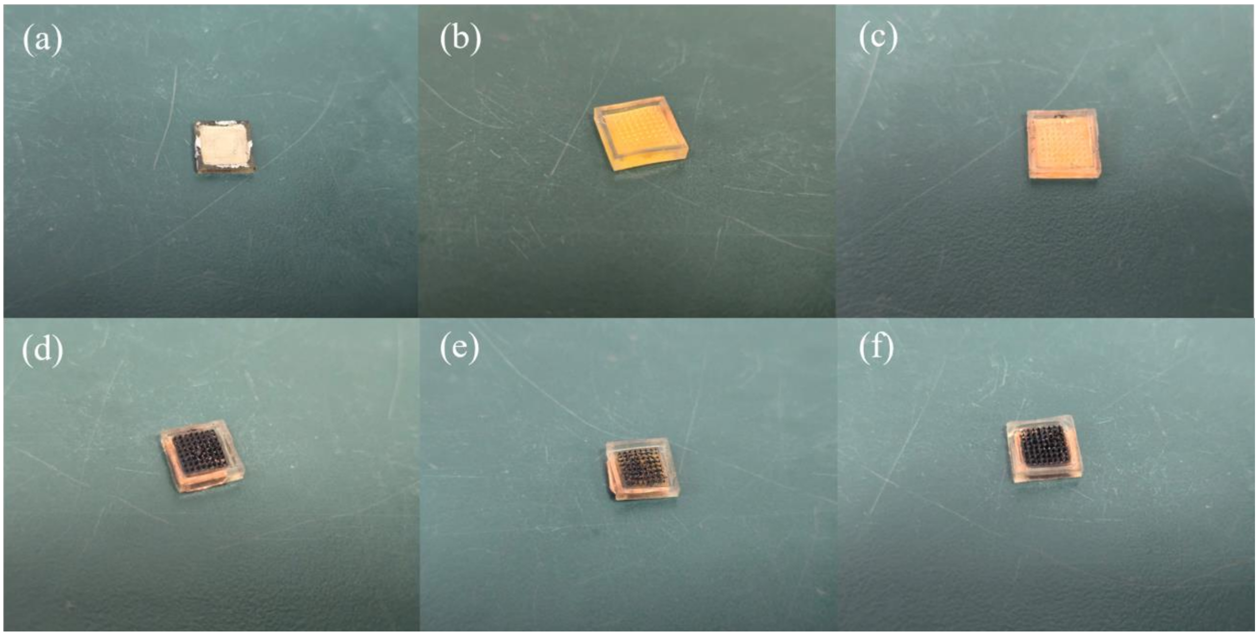

Figure S3. The working electrode and reference electrode of the sensor prepared for each sample
(a) reference electrode; (b) working electrodes of enzyme-containing microneedle sensors; (c) Coated with the working electrode of the GED enzyme microneedle sensor; (d) Coated with polyaniline microneedle sensor working electrode; (e) Coated with polyaniline-containing enzyme microneedle sensor working electrode; (f) Coated with GED enzyme and polyaniline microneedle sensor working electrodes

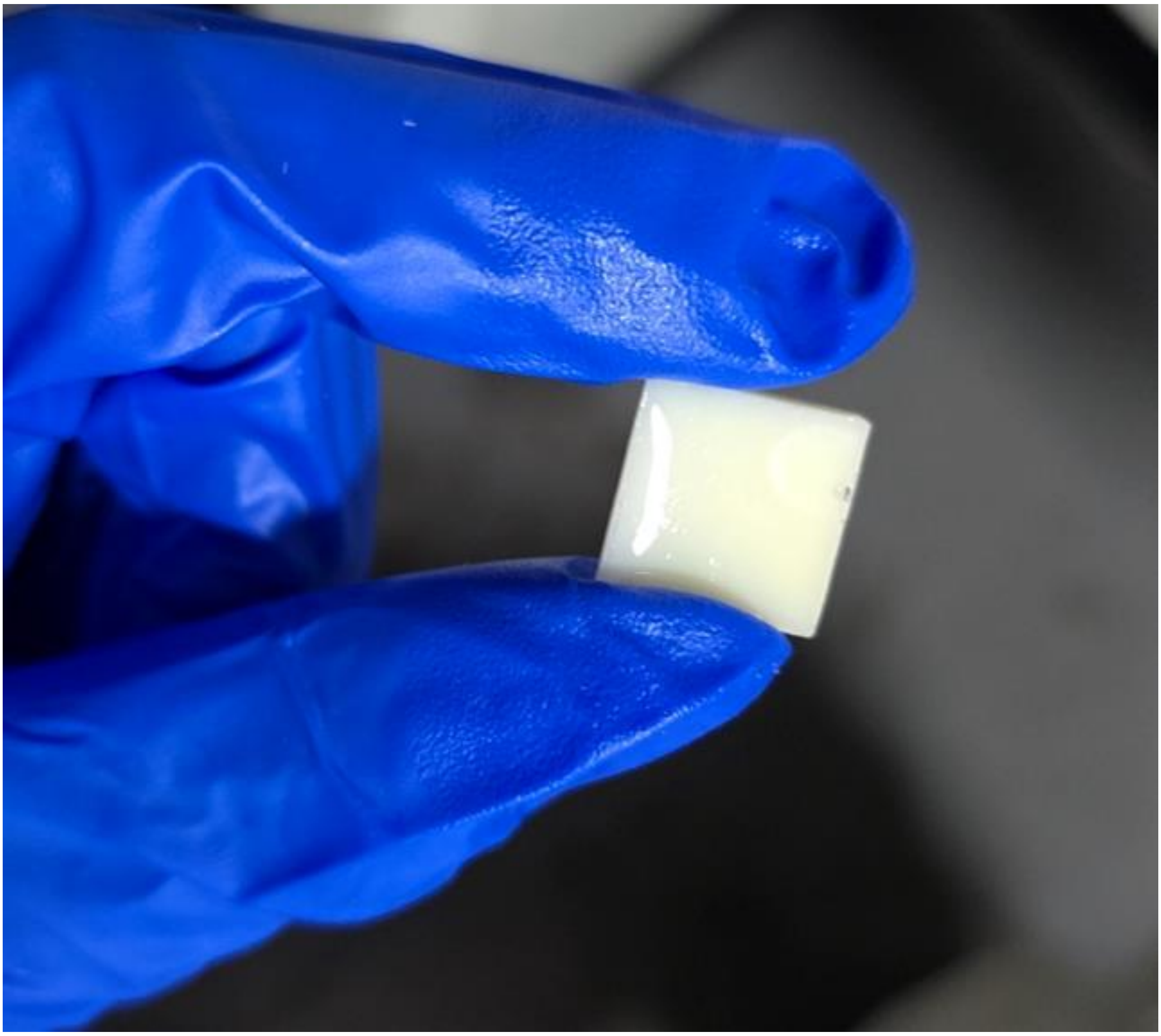
Figure S4. Ionic gels with obvious phase separation (IPDI:PCL:PEG=11:1:8, 11:0.5:8, 11:0.5:7)

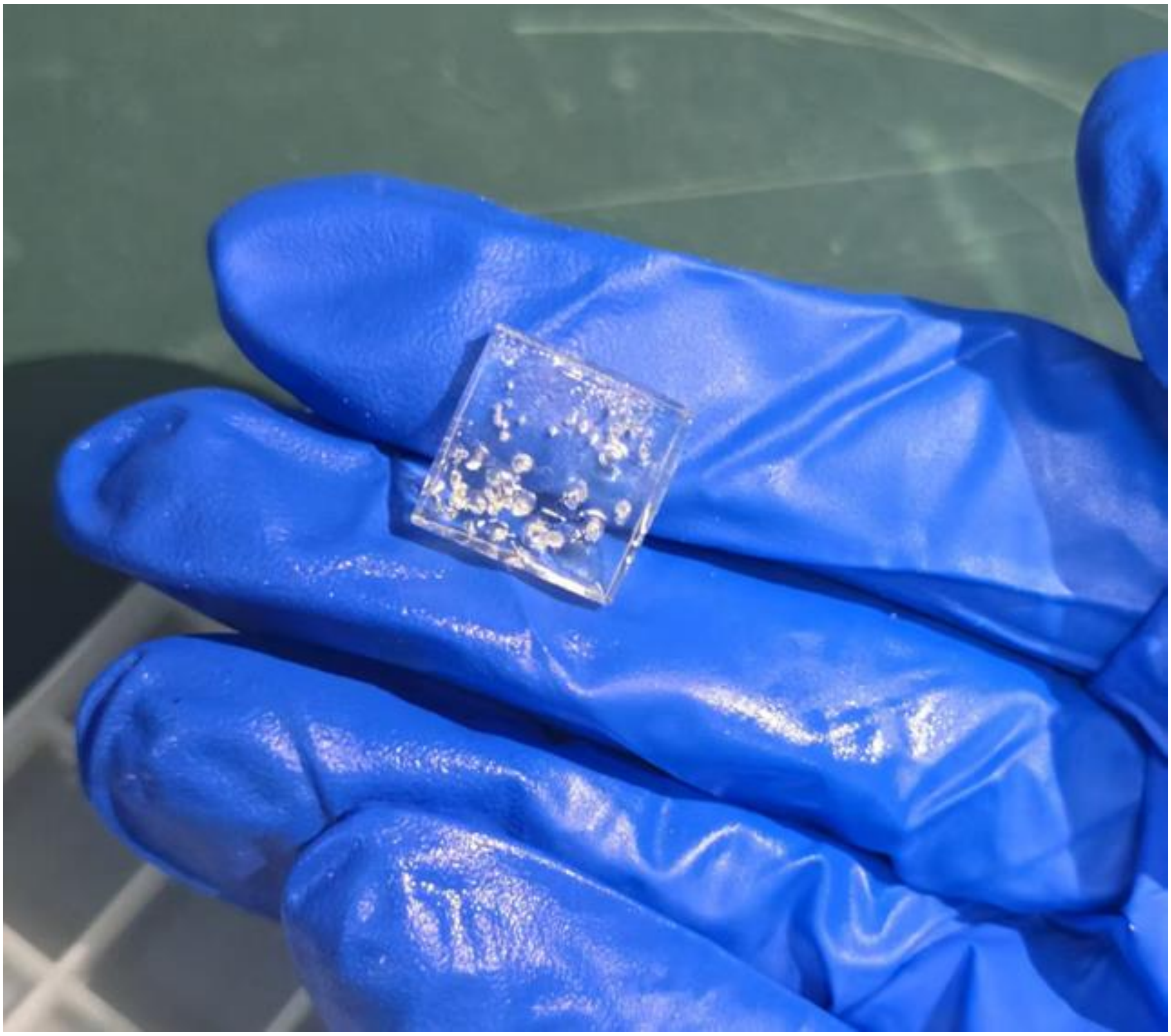

Figure S5. Ionic gels without phase separation (IPDI:PCL:PEG=11:0.5:6, 11:0.5:5.5, 11:0.5:5)

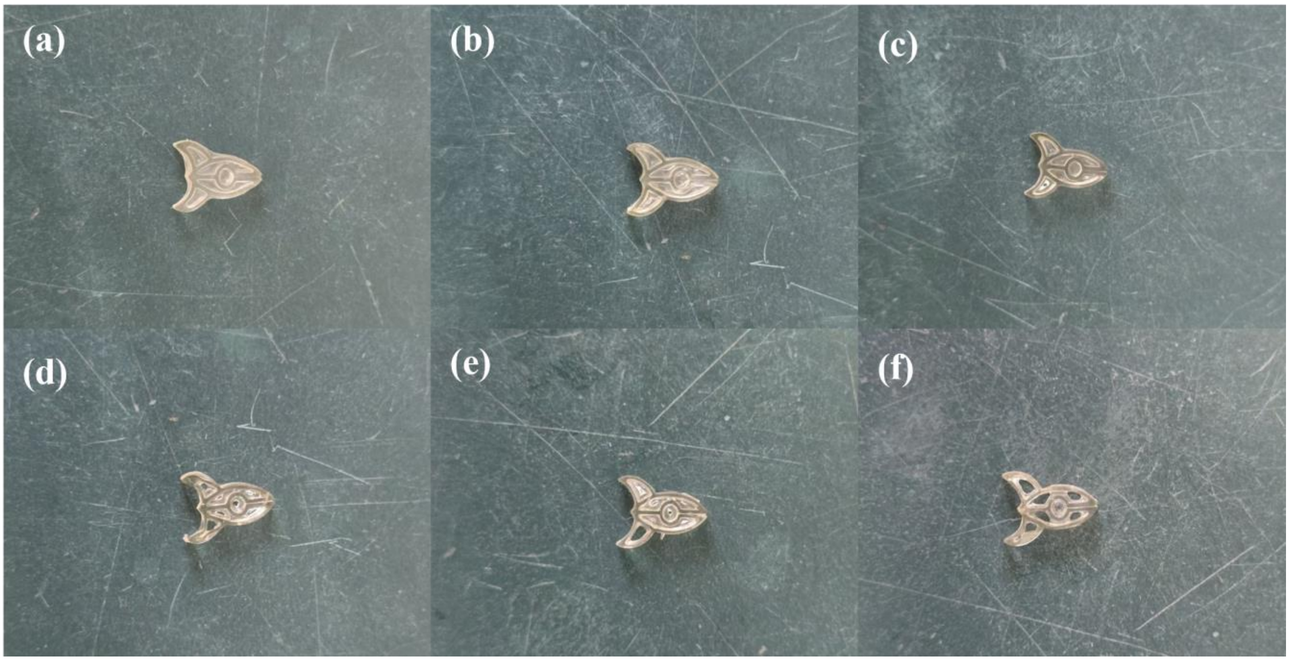

Figure S6. Comparison of hollow rocket models with different parameters
(a) Finished model with light intensity of 8 mW/cm-2 and time of 12 s; (b) Finished model with light intensity of 8 mW/cm-2 and time of 10 s; (c) Finished product of the model under the parameters of light intensity of 8 mW/cm-2 and time of 6 s; (a) Finished product of the model under the parameters of light intensity of 8 mW/cm-2 and time of 4 s; (d) The finished model under the parameters of light intensity 6 mW/cm-2 and time 6 s; (e) The finished product of the model with a light intensity of 4 mW/cm-2 and a time of 6 s

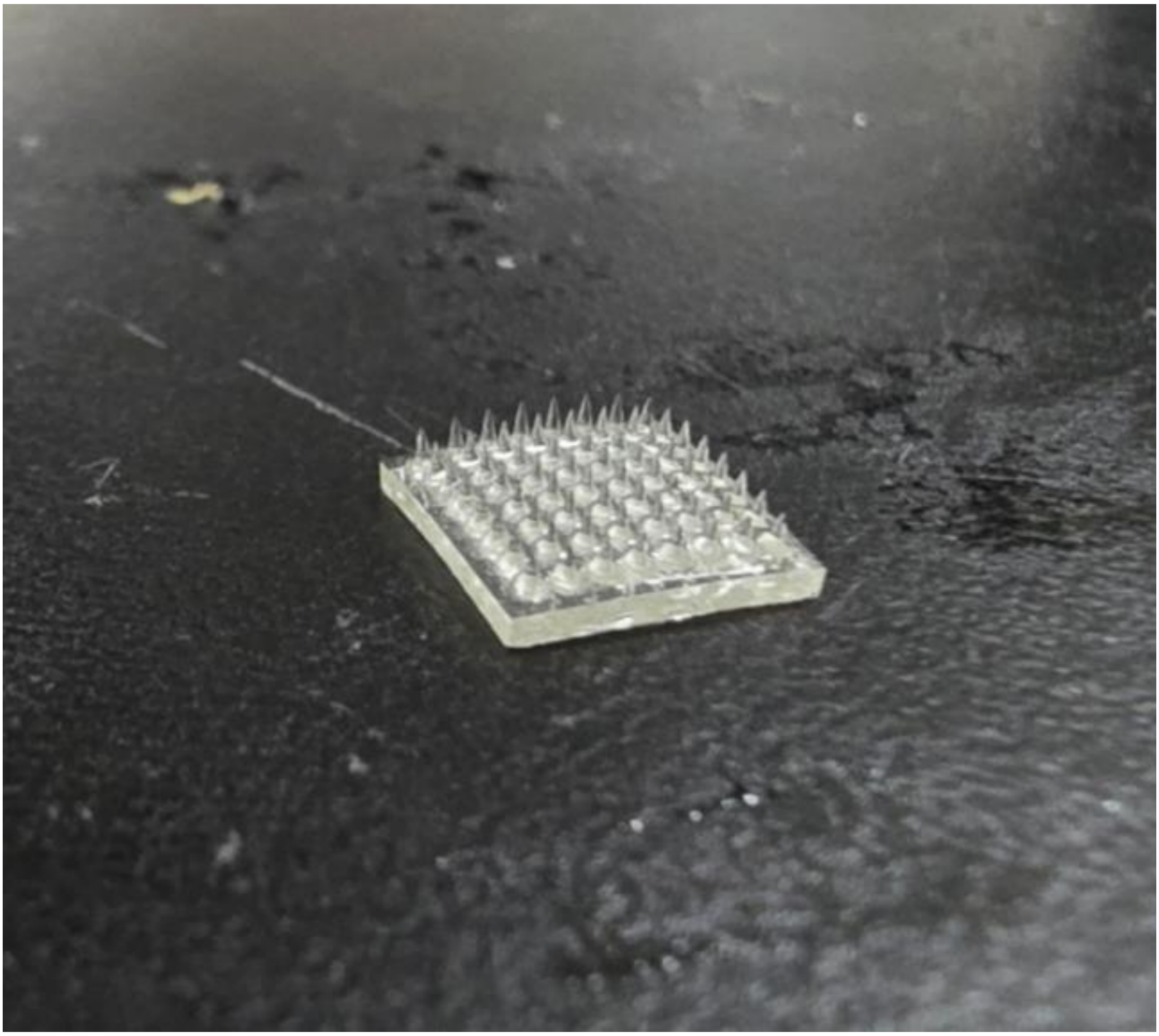

Figure S7. Actual drawing of the microneedle model

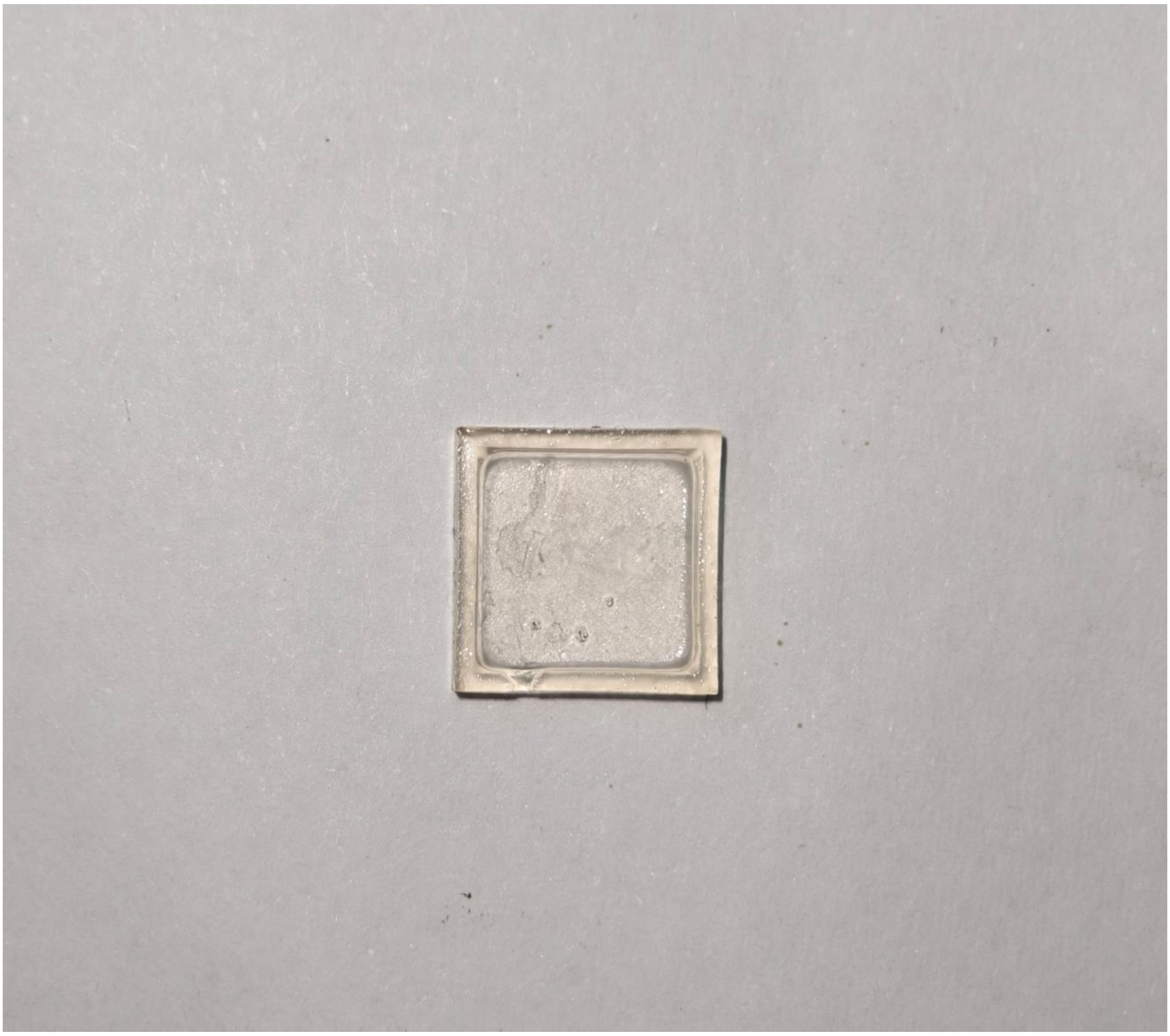

Figure S8. Actual drawing of the groove model
